# The *Turnera* Style *S*-Locus Gene *TsBAHD* Possesses Brassinosteroid-Inactivating Activity When Expressed in *Arabidopsis thaliana*

**DOI:** 10.3390/plants9111566

**Published:** 2020-11-13

**Authors:** Courtney M. Matzke, Joel S. Shore, Michael M. Neff, Andrew G. McCubbin

**Affiliations:** 1 School of Biological Sciences, Washington State University, PO Box 644236, Pullman, WA 99164-4236, USA; courtney.matzke@wsu.edu; 2 Department of Biology, York University, 4700 Keele Street, Toronto, ON M3J1P3, Canada; shore@yorku.ca; 3 Department of Crops and Soils, Washington State University, PO Box 644236, Pullman, WA 99164, USA; mmneff@wsu.edu

**Keywords:** heterostyly, BAHD acyltransferase

## Abstract

Heterostyly distinct hermaphroditic floral morphs enforce outbreeding. Morphs differ structurally, promote cross-pollination, and physiologically block self-fertilization. In *Turnera* the self-incompatibility (S)-locus controlling heterostyly possesses three genes specific to short-styled morph genomes. Only one gene, *TsBAHD*, is expressed in pistils and this has been hypothesized to possess brassinosteroid (BR)-inactivating activity. We tested this hypothesis using heterologous expression in *Arabidopsis thaliana* as a bioassay, thereby assessing growth phenotype, and the impacts on the expression of endogenous genes involved in BR homeostasis and seedling photomorphogenesis. Transgenic *A. thaliana* expressing *TsBAHD* displayed phenotypes typical of BR-deficient mutants, with phenotype severity dependent on *TsBAHD* expression level. *BAS1*, which encodes an enzyme involved in BR inactivation, was downregulated in *TsBAHD*-expressing lines. *CPD* and *DWF,* which encode enzymes involved in BR biosynthesis, were upregulated. Hypocotyl growth of *TsBAHD* dwarfs responded to application of brassinolide in light and dark in a manner typical of plants over-expressing genes encoding BR-inactivating activity. These results provide empirical support for the hypothesis that TsBAHD possesses BR-inactivating activity. Further this suggests that style length in *Turnera* is controlled by the same mechanism (BR inactivation) as that reported for *Primula*, but using a different class of enzyme. This reveals interesting convergent evolution in a biochemical mechanism to regulate floral form in heterostyly.

## 1. Introduction

**Figure 1:**
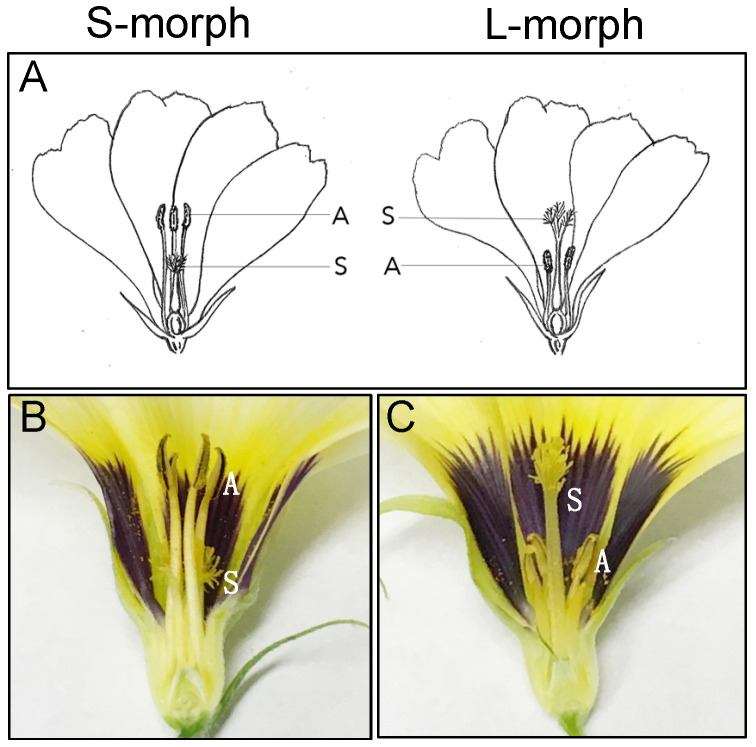
Phenotypes of short (S-morph) and long (L-morph) style morphs. (**A**) Diagram showing reciprocal arrangement of stigma and style (S) and anthers and stamens (A). (**B**,**C**) Longitudinal section of a mature flower showing the short morph and long morph of *Turnera subulata*.

Most flowering plants bear flowers with male and female reproductive structures in close physical proximity. Logically this should lead to an inherent tendency to inbreed, but most angiosperm species have evolved mechanisms that promote outbreeding and/or prevent self-fertilization [1,2]. Such breeding systems help to maintain genetic diversity within species, a key factor for long term evolutionary success. In species that possess heterostyly, individual plants bear flowers of one of two (distyly) or three (tristyly) floral morphs, between which male and female organs are reciprocally positioned, promoting cross-pollination (Figure 1). These morphological differences are often combined with within-morph biochemical incompatibility, forming a breeding system that acts to prevent self- and intra-morph fertilization in addition to promoting out-crossing. The significance and widespread polyphyletic occurrence of heterostyly has garnered the attentions of many researchers seeking to unravel the evolutionary and genetic mechanisms of the polymorphism. 

Darwin was intrigued by this phenomenon, and after extensive studies in *Primula* was the first to conclude that the significance of the floral forms lies in contributing to outcrossing [3]. Being a trait readily scored by eye, heterostyly was a popular model in the early efforts to demonstrate single trait Mendelian inheritance [1]. Later the discovery of rare natural homostyle individuals, which bear flowers in which stamen and stigma are at the same level, generated further interest. Subsequent analyses led to the proposition that heterostyly is controlled by a supergene locus, the “*S*-locus,” comprised of several tightly linked genes with recombination between alleles occurring only very rarely, but leading to homostyly [4]. Such interpretations inferred the short styled (S)-morph to be heterozygous for the *S*-locus, *Ss*, and the long styled (L)-morph homozygous recessive, *ss* [5,6]. As hemizygous (see below) supergene loci involved in controlling mating type, *S*-loci controlling heterostyly are far from unique; indeed, they share functional and physical commonalities with sex chromosomes (the *S*-locus-bearing chromosome is akin to the Y chromosome), albeit that in heterostyly mating types retain hermaphroditism. The genes that control these breeding barriers are of interest as potential tools with which to engineer new or improved barriers in and between agricultural species and their relatives.

In recent years, genes within the *S*-locus that control distyly have been identified in several unrelated genera, including *Primula*, *Turnera*, and *Fagopyrum* (see [7] for review). Surprisingly, though these genera appear to have independently evolved heterostyly, genetically their *S*-loci all appear to function in a manner contrary to the historical dogma. Rather than there being dominant and recessive *S*-alleles as previously accepted, the genes are in fact hemizygous, being present in the S-morph, but lacking allelic counterparts in the L-morph [8,9,10,11]. These *S*-genes, their roles in heterostyly, and their modes of action are focal points of current research.

In *Primula*, five genes have been identified within the *S*-locus: *CYP734A50* (a cytochrome p450), GLOBOSA2 (*GLO2*, a MADS box transcription factor), *PUM* (a Pumilio-like RNA-binding protein), *CCM* (a cyclin-like F box gene), and *KFB* (a Kelch repeat F box protein) [10,12,13,14,15]. *CYP734A50* is a member of the P450 CYP734A cytochrome family expressed in pistils [13]. The closest *Arabidopsis thaliana* homolog of *CYP734A50* is *PHYB ACTIVATION-TAGGED SUPRESSOR1* (*BAS1*), a gene that encodes an enzyme with BR degrading activity [13,16]. This observation led to an elegant set of experiments that demonstrated that *Primula CYP734A50* controls pistil length in the S-morph by reducing cell expansion by degrading BRs [13]. *GLOBOSA2* (*GLO2)* is a MADS-box transcription factor expressed specifically in S-morph stamens [10,12,15], and was recently shown to control (elevate) anther height by promoting cell expansion in the corolla tube below the point of anther filament insertion [14]. The potential functions of the other three *Primula S*-genes are as yet unknown. In *Fagopyrum*, style length is regulated by *S*-locus early flowering 3 (*S-ELF3*), a homolog of *A. thaliana ELF3*, a nuclear protein that interacts with phytochrome B to control plant development and flowering [8,9].

The *Turnera S*-locus possesses only three genes. Based on homology to known gene families, they have been named *TsSPH1* (homologous to *Papaver rhoeas* stigma incompatibility *S-protein*), *TsYUC6* (homologous to the *YUCCA* gene family of flavin-dependent monooxygenases), and *TsBAHD* (homologous to the BAHD acyltransferase family) [11]. *TsSPH1* is expressed in filaments and anthers, and may be involved in controlling filament length [11]. *TsYUC6*, is expressed in anthers, potentially involved in auxin synthesis, and likely to control pollen incompatibility mating type [11,17,18]. The third gene, *TsBAHD*, has conserved motifs found in BAHD acyltransferases and is the only *S*-gene expressed in pistils, necessitating that it controls all female characteristics, including style length. 

The closest characterized *A. thaliana* homologs of *TsBAHD* include genes reported to acetylate and inactivate BRs [19]. This leads us to hypothesize that TsBAHD causes short styles in *Turnera* by acetylating and inactivating BRs, employing the same mechanism as that found in *Primula* but effected by a different class of BR-inactivating enzyme [11,13]. Support for this hypothesis has been found in the form of patterns of BR regulated gene expression in transcriptome analyses, where depletion of BR regulated genes was found in S-morph relative to L-morph pistils [20]. However, an empirical test of this hypothesized activity is lacking, but necessary. The BAHD family is a large gene family (with 64 members in *A. thaliana* alone) that consists of acyltransferases that catalyze transfer of acyl-groups from CoA thioester compounds to several different types of plant metabolites [21]. BAHD is an acronym composed of the first four active enzymes that were classified biochemically, BEAT (benzyl alcohol *O*-acetyltransferase from *Clarkia breweri*), AHCT (anthocyanin *O*-hydroxycinnamoyltransferase from *Gentiana triflora*), HCBT (N-hydroxycinnamoyl/benzoyltransferase from *Dianthus caryophyllus*), and DAT (deacetylvindoline 4-*O*-acetyltransferase from *Catharanthus roseus*) [22]. The majority of BAHD genes have not been characterized, and though it is reasonable to assume based on conserved sequence motifs, that *TsBAHD* is in the BAHD protein family, inferring biochemical function by sequence homology alone has proven difficult in this family [23]. Amino acid alignments used to create phylogenetic trees separate BAHD family members into five different clades predicted to share either substrate preference or conditions for activity [23,24,25]. Even though these analyses may place a gene within a certain clade, characteristics such as substrate preference and function are not always similar [23]. Hence additional work was required to further biochemically characterize *TsBAHD*.

BRs play several roles in plant development, including promoting cell elongation [26]. BRs are found at very low concentrations in plant tissues and cannot be transported extracellularly [27], their homeostasis is tightly regulated by negative feedback loops and multiple mechanisms of inactivation [28]. *A. thaliana* plants mutated to prevent synthesis of the bioactive BR brassinolide (BL) exhibit severe dwarf phenotypes typically with shortened stems and petioles, shortened, epinastic, dark green leaves, delayed senescence, and infertility (for review see [26]). Importantly, these phenotypes can be rescued by application of exogenous BL [29,30,31]. 

Several BR biosynthetic genes have been identified, including *DE-ETIOLATED2* (*DET2*) [29], DWARF4 (*DWF4)* [28], and *CONSTITUTIVE PHOTOMORPHGENESIS AND DWARFISM (CPD*) [30], and these are upregulated in *A. thaliana* plants with low levels of BR [32]. To maintain BR homeostasis, catabolic enzymes are also dynamically expressed and are active along several parts of the BR pathway to degrade or inactivate BR. Increased expression of these catabolic enzymes leads to reduced BR and characteristics typical of BR-deficient dwarfs. For example, over-expression of *BAS1*, which encodes an enzyme that inactivates BRs by hydroxylation, results in shortened hypocotyls [16,33]. Members of the BAHD acyltransferase family, including *BRASSINOSTEROID INACTIVATOR1* (*BIA1)* and *BIA2*, have been also been assessed using over-expression bioassays in *A. thaliana*, and have been reported to possess BR inactivation activity effected by acyl conjugation of BRs [19,34]. 

In this study, we investigated whether *TsBAHD* encodes, as hypothesized, a BR degrading acyltransferase [11], by employing a bioassay in which *TsBAHD* was over-expressed in *A. thaliana*. We show that *TsBAHD* caused dwarf phenotypes consistent with BR-deficiency. These dwarfs exhibited upregulation of genes encoding classic BR biosynthesis enzymes, *DWF4* and *CPD,* and downregulation of a gene encoding the BR degrading enzyme, *BAS1*. Exogenous BL application led to increased hypocotyl growth in *35S::TsBAHD* dwarfs similar to that seen in wild type plants when grown in the light. In contrast, very little growth occurred in BL treated *35S::TsBAHD* lines when grown in the dark. These data provide empirical evidence consistent with TsBAHD possessing BR-inactivating enzyme activity, and further suggest this activity involves acylation of BL rather than targeting earlier intermediates in the BR synthesis pathway.

## 2. Results

### 2.1. Expression of TsBAHD in A. Thaliana Induces Dwarfism

**Figure 2:**
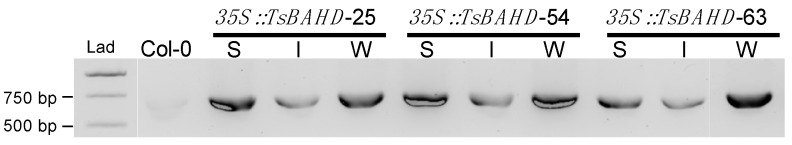
Confirmation of insert in transgenic lines in *A. thaliana*. Three independent lines of the *TsBAHD* overexpression construct. Each *35S::TsBAHD* line has been confirmed in severe (S), intermediate (I), and weak (W) dwarf phenotypes, represented by the three bands under each *35S::TsBAHD* line in corresponding order. Expected fragment size is 683 bp. Each lane contained 5 µL of PCR product and 2 µL of loading dye. LAD was 5 µL of a 1 kb DNA ladder.

**Figure 3:**
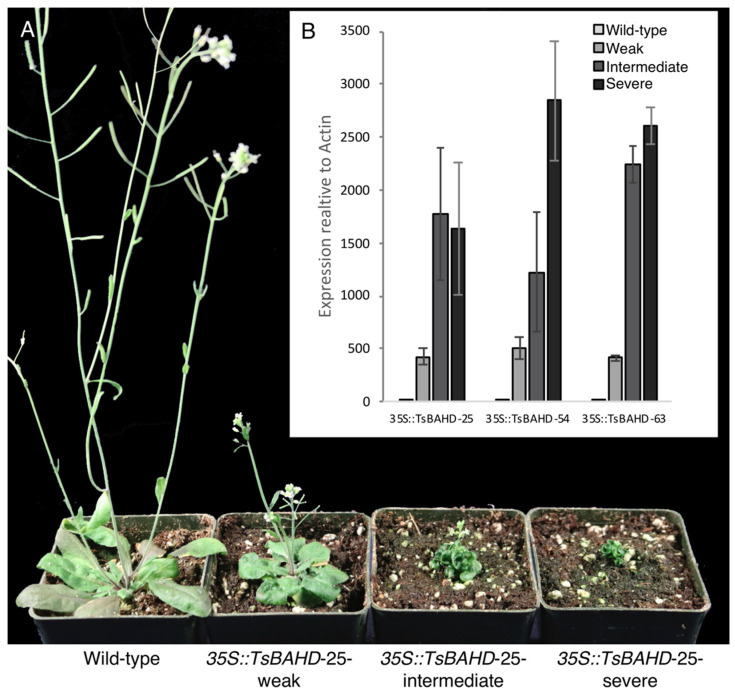
Phenotypes and expression of *35S::TsBAHD*-25 in *A. thaliana* normalized to the actin 8 (At1g4920) housekeeping gene. (**A**) From left to right: six week old plants of wild type (Col-0) compared to three *35S::TsBAHD* (line 25) transformants showing weak, intermediate, and most severe dwarf phenotypes. (**B**) Expression of *35S::TsBAHD* lines 25, 54, and 63 in S, I, and W dwarfs compared to the wild type. Wild-type samples were included as a negative control. Errors bars represent standard error.

*A. thaliana* plants were transformed with *35S::TsBAHD* by floral dip [35], using *Agrobacterium* strain GV 31010 [36]. Transformants were initially identified by kanamycin selection and the presence of the transgene confirmed by PCR (Figure 2). Independent lines were selected, assessed to possess single locus insertions based on segregation of kanamycin resistance, and three independent homozygous lines (*35S::TsBAHD* lines 25, 54, and 63) were generated. Though all progeny for all three lines displayed dwarf phenotypes, within all lines the severity of dwarfism varied between individuals. To facilitate further investigation, plants were selected representing severe (S), intermediate (I), or weak (W) dwarf phenotypes (Figure 3A). Severe dwarfs were extremely reduced in stature with very small, epinastic, dark green leaves, and reduced inflorescence that set very little seed on self-fertilization. Intermediate dwarfs were similar to but somewhat larger than S dwarfs, and had longer flower stalks and higher self-fertility. Weak dwarfs had larger, lighter green leaves and longer stems, but were still substantially reduced in size relative to wild type. Fertility in the weak dwarfs was also closer to wild type, although the flower stalks were still much shorter than those of wild-type plants (Figure 3A). These phenotypes are consistent with those reported for BR-deficient mutants, including *DET2*, *DWF4*, and *BAS1* [16,28,29,30]. We reasoned that the variations in phenotype might relate to expression level of the transgene caused by variable transgene silencing. This hypothesis was tested using qPCR to assess *TsBAHD* expression in S, I, and W dwarfs of all three lines. This analysis showed that the severity of dwarfism correlated well with *TsBAHD* expression in the transgenic lines. Expression was consistently lowest in W dwarfs and higher in I and S dwarfs, with S dwarfs exhibiting the highest expression levels in *35S::TsBAHD* lines 54 and 63, though not significantly different from I dwarfs in line 25 (Figure 3B). These results are broadly consistent with *TsBAHD* expression inducing the observed dwarfism, leading us to investigate whether this dwarfism was mediated by BR degradation.

### 2.2. Assessment of Expression of BR Biosynthesis and Inactivating Genes 

**Figure 4:**
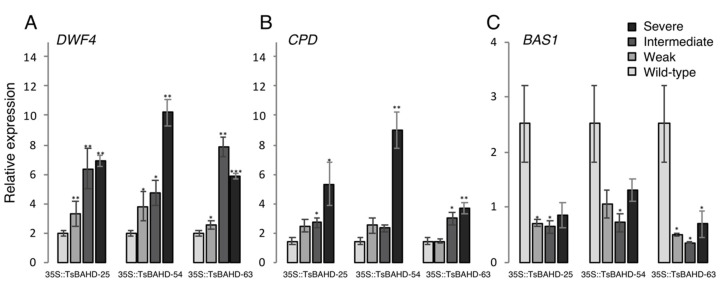
Relative expression of BR biosynthesis and inactivating genes in *35S::TsBAHD* normalized to an actin housekeeping gene. Expressions of *DWF4* (**A**), *CPD* (**B**), and *BAS1* (**C**) in six week old *35S::TsBAHD* lines 24, 54, and 63. All *35S::TsBAHD* transformants showing S, I, and W phenotypes compared to the Col-0 wild type. Each qRT-PCR value is the mean of three replicates. Errors bars represent standard error. *p*-values comparing *35S::TsBAHD* lines to the wild type were calculated using Student’s *t*-test comparing each line to the wild-type expression * *p* < 0.05, ** *p* < 0.01, *** *p* < 0.001.

BR homeostasis is maintained by feedback regulation, mediated by variable expression of a number of genes [26]. Relative to wild-type plants, BR-deficient dwarfs typically display up-regulation of genes involved in BR biosynthesis and down-regulation of those involved in BR inactivation [32,37,38]. To assess whether the dwarf phenotypes were associated with altered BR signaling, expressions of several well characterized genes related to BR biosynthesis and inactivation were measured (Figure 4). Two canonical genes that encode enzymes involved in BR biosynthesis, *DWF4* and *CPD*, showed elevated expression in all dwarf phenotypes relative to wild-type plants (Figure 4A,B). S dwarfs showed the largest increase in *CPD* expression, exhibiting statistically significant 2–3 fold increases in the three independent lines when compared to the wild type (*p*-values < 0.01). I dwarfs exhibited significant 1–2 fold increases in two lines (*p*-values 0.001 and 0.002), and an increase in the third line that was not statistically significant. W dwarfs also showed significant increases compared to wild type in two lines (*p*-values 0.002 and 0.04). Expression of *DWF4* relative to the wild type showed a 2–3 fold increase in S dwarfs (*p*-values 0.001 and 0.04). I and W dwarfs showed smaller, less statistically significant increases (*p*-value 0.01). *BAS1*, which encodes a BR-inactivating enzyme, showed lowered expression relative to wild type in all three dwarf phenotypes (Figure 4C). I and W dwarfs showed largest and the most significant decreases with almost 3-fold reductions (*p*-values 0.02–0.04). S dwarfs showed an about 2 fold decrease with a significant *p*-value in one line (0.04). These patterns of gene regulation in the *35S::TsBAHD* lines are consistent with those that would be expected if altered BR levels underpin the dwarf phenotypes. We next sought to determine if application of exogenous BR could rescue *35S:TsBAHD* induced dwarfism.

### 2.3. Response of TsBAHD Expressing Hypocotyls to Exogenous BR in Light and Dark

**Figure 5:**
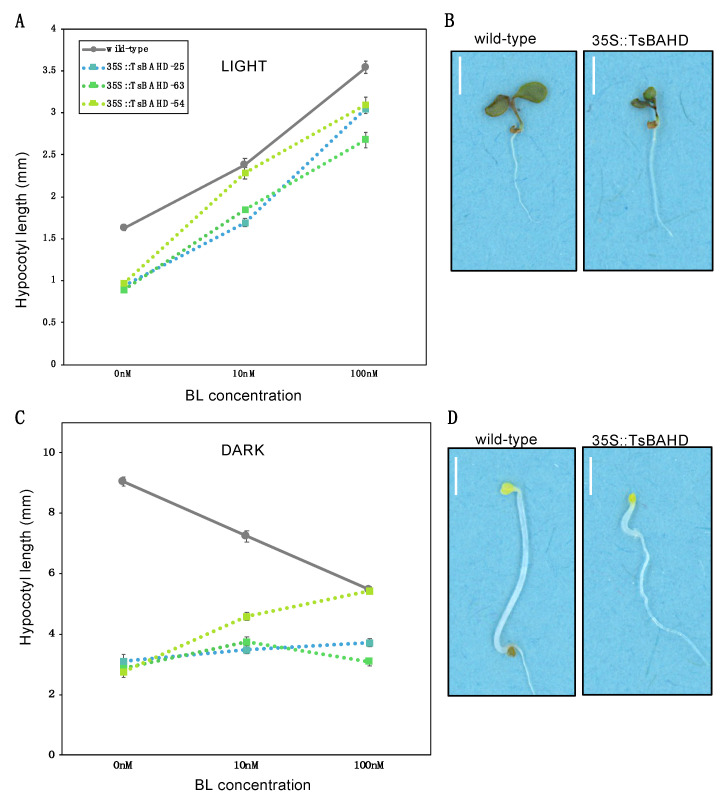
Hypocotyl response to different concentrations of brassinolide (BL) in light (**A**,**B**) and dark (**C**,**D**). Seedlings pictured are representatives of wild type and *35S::TsBAHD* lines from plates with 0 nM BL. Hypocotyls of 4 day old seedlings were grown on plates at 25 °C in continuous light at an intensity of 80 µE and in complete darkness. Plates contained half strength Linsmaier and Skoog modified basal medium, 0.6% Gellan, and 1.5% sucrose. BL concentrations were 0, 10, and 100 nM. Green dotted lines represent two replicates of three independent lines of *35S::TsBAHD* and solid gray line represents the wild type. Errors bars represent standard error. Scale bars = 2 mm.

To determine if dwarfism in *35S::TsBAHD* transgenic lines could be rescued by addition of BRs, dwarfs were grown in increasing concentrations of BL under different light conditions and hypocotyl growth was measured. If the TsBAHD protein is involved in BR inactivation, in *35S::TsBAHD* hypocotyls are expected to be shorter than those of the wild type when grown in the light, and exhibit a de-etiolated hypocotyl phenotype when grown in the dark. In the light in the absence of BL, transgenic lines exhibited significantly shorter hypocotyl lengths (mean = 0.93 mm; S.E. = 0.03) than the wild type (mean = 1.6 mm, S.E. = 0.03) (Figure 5A,B). At 10 nM BL, wild type and dwarf hypocotyls increased in length, and by 100 nM, the lengths of the wild type (mean = 3.54 mm, S.E. = 0.07) and all three lines of *35S::TsBAHD* (mean, 3.04 mm; S.E. = 0.52) had significantly longer hypocotyls relative to their lengths at 0 nM. Two of the *35S::TsBAHD* lines increased in hypocotyl length in response to increasing BL concentration at approximately the same rate as the wild type but remained shorter. One line, however, *35S::TsBAHD*-54, showed a greater response to BL and reached a similar length (mean = 2.2 mm) to the wild type (mean, 2.1 mm) by 10 nM BL. Analysis by two-way ANOVA revealed marked phenotype effects (F2,45 = 25.5, *p*-value ≤ 0.001), no strain effects (F2,45 = 2.64, *p*-value = 0.08), and a marginally significant interaction (F4,45 = 2.74, *p*-value = 0.04).

In contrast, when grown in the dark in the absence of BL, *35S::TsBAHD* seedlings had drastically shorter hypocotyls (average 2.6 mm) than the etiolated wild type plants grown on the same plate (9.1 mm). As the concentration of exogenous BL applied increased, wild type hypocotyl length severely decreased (from 9.05 mm S.E. = 0.13, to 5.5 mm S.E. = 0.08). On average, the *35S::TsBAHD* lines showed a small gradual increase if any change was observed (Figure 5C,D). Between 10 and 100 nM, two of the *35S::TsBAHD* lines remained the same or showed a minor decrease. The third line *35S::TsBAHD*-54 showed a small increase in hypocotyl length (from 3.8 to 5.2 mm).

## 3. Discussion

In this study, the mechanism controlling heterostyly in *Turnera* was investigated. *TsBAHD* was recently identified as the G (gynoecium) component of the *S*-locus in *Turnera* that determines this trait [11]. The *S*-locus in *Turnera* is hemizygous, being present as a single copy in the short style morph, and absent from the long-styled morph, as is also the case for the *S*-locus of *Primula* [11]. In *Primula* the *S*-locus gene controlling style length is *CYP734A50*, a BR degrading enzyme with homology to *BAS1*, which degrades BRs in *A. thaliana*. *TsBAHD* in contrast shares homology with BAHD acyltransferases, as some members of this gene family acetylate and inactivate BRs. We previously hypothesized that *TsBAHD* may also be capable of inactivating BRs [11]. If this is the case it would suggest convergent evolution between the *S*-loci of *Turnera* and *Primula*, such that different BR-inactivating different genes have been recruited to the *S*-locus to cause shortened style length. To investigate this possibility and the biochemical function of *TsBAHD*, the gene was expressed in *A. thaliana*. 

*A. thaliana* plants expressing *TsBAHD* displayed dwarf phenotypes typical of BR-deficient mutants including small, epinastic, dark green leaves, shortened stems, and self-infertility. The severity of dwarfism was variable but correlated with the expression level of *TsBAHD*, consistent with the transgene being responsible for the phenotype. Transgene suppression is a likely cause of the phenotype variability. Due to the very low levels of BRs present in plants and the lack of long-distance transport, the homeostasis of precursors and intermediates in the BR pathway is tightly regulated [39]. When BR levels approach either low or high levels, the expression of genes involved in the regulatory pathway changes, leading to either synthesis or inactivation of BRs. To determine if expression of *TsBAHD* had perturbed expression of genes involved in BR homeostasis, we assessed the expression of two genes involved in BR biosynthesis (*DWF4* and *CPD*), and one in BR inactivation (*BAS1*) in the transgenic plants relative to wild type plants. The expression of *DWF4* and *CPD* increased with increased transgene expression, whereas *BAS1* decreased, consistent with *TsBAHD* expression, leading to reduced BR levels in the transgenic lines. These results are consistent with a negative feedback response resulting from low BRs concentrations within plants expressing *TsBAHD*.

Studying the effect of light, in combination with BR treatment on hypocotyl growth, was also informative. BRs are important in photomorphogenesis, and so have major impacts on growth and development in a light dependent manner [29,30,40]. When grown in the light, the hypocotyl length of *35S::TsBAHD* lines was significantly reduced compared to wild type, which is a characteristic of BR mutants. When germinated in the dark *35S::TsBAHD* seedlings showed a severely reduced etiolation response or lacked one entirely. The results of this study are consistent with previous work on BR-deficient dwarfs grown in light or complete darkness [16,19,40,41]. Additional information as to which form of BR may be the substrate of this enzyme was gleaned by assessing the ability of BL to rescue *TsBAHD* induced dwarfism. In the light, as BL was added in increasing concentrations up to 100 nM, *35S::TsBAHD* hypocotyls increased in length at approximately the same rate and proportion as wild type, showing that exogenously applied BL was not able to fully rescue the phenotype. Conversely, when grown in the dark with BL, a subtle decrease in hypocotyl length or lack of effect was observed for the *35S::TsBAHD* lines. Again these results mirror those previously reported in several BL dose response experiments in which BR degrading genes were overexpressed [16,34,41,42]. The drastic decrease observed in hypocotyl length of wild type seedlings when grown in increasing amounts of BL in the dark may be due to toxicity of the excess amount of BL suddenly present. As BR is usually present in small quantities within plant tissues, overabundance may halt and decrease development as the plant is unable to process the excess. Alternatively, it may be that in the dark, since BL is the endpoint of the pathway, exogenous BL application is not sufficient to signal activation of the degradation pathway [37]. The results provide clues as to which intermediate in the BR pathway *TsBAHD* may be acting upon. If the substrate for TsBAHD is later in the pathway, such as the end product BL, exogenously applied BL would be immediately inactivated, and so have little effect on hypocotyl length. If instead the substrate for TsBAHD is a precursor or intermediate(s) earlier on in the pathway, then the expected result would be reestablishment of active BL and other products further downstream. The lack of ability of BL to rescue the phenotypes observed in this study suggests that the preferred substrate for the TsBAHD enzyme is an end product of the BR biosynthesis pathway, possibly BL itself. 

Together, the results presented in this study are consistent with overexpression of *TsBAHD* leading to dwarf phenotypes resulting from reduced BR levels, and provide substantial empirical support for the hypothesis that *TsBAHD* encodes a BR-inactivating enzyme. As *TsBAHD* is the only *S*-locus gene expressed in pistils in *Turnera subulate*; this suggests that style length in this species is controlled by the same mechanism (BR inactivation) as that reported in *Primula* [13], albeit using different genes encoding distinct enzyme activities. Hence a novel example of convergent evolution in a biochemical mechanism to regulate floral form in heterostyly has been revealed. As this mechanism appears to have evolved twice to generate a breeding barrier in distinct species, it is tempting to speculate that it may be a pathway through which flower morphology and/or breeding barriers might be engineered in other plant species.

## 4. Materials and Methods 

### 4.1. Plasmid Construction and Generation of Transgenic Lines

The polymerase chain reaction (PCR) was used to amplify the *TsBAHD* coding sequence (GenBank: MK922466.1) from a bacterial artificial chromosome clone, BAC-A24 (11) using Accuzyme™ proofreading Taq DNA polymerase (Bioline, USA). Gene-specific primers created for amplification were (5′-TTAAGATATGGAAGTTGAGAT-3′ and 5′-CAAAAGCATGATTCTG-3′) at 58 °C annealing temperature. The cloned gene was ligated into pGEM-T^®^-T Easy vector (Promega, USA). DNA was purified using the ZR Plasmid Miniprep-Classic and Zymoclean™ Gel DNA Recovery Kit according to the manufacturers’ instructions (ZymoResearch, USA) and sequenced. *Bam*H1 and *Kpn*1 restriction sites were added using primers (5′-GGTACCTTAAGATATGGAAGTTGAGAT-3′ and 5′-GGATCCCAAAAGCATGATTCTG-3′) to facilitate cloning behind the cauliflower mosaic virus (CaMV) 35S promoter in the binary vector pCHF3 [16]. After verifying the insert by Sanger sequencing, transformation constructs were then transformed into *Agrobacterium* strain GV3101 and the floral dip method used to transform the *A. thaliana* ecotype Col-0 [35].

### 4.2. Plant Growth Conditions and Transformation Screening 

In all experiments, *A. thaliana* seeds were surface sterilized by shaking for 15 min at room temperature in a solution of 70% ethanol, 0.05% Triton X 100. This was repeated with 95% ethanol, 0.05% Triton X 100 followed by a rinse with 95% ethanol. Seedlings were grown on half strength Linsmaier and Skoog modified basal medium, 0.8% Phytoblend (Caisson Laboratories, Smithfield, UT, USA), and 1.5% sucrose. Plates were placed in a cold, dark environment (4 °C) for 3 days to promote even germination. Growth chamber conditions for all seedlings (unless otherwise noted) were 100 µmol m^−2^ s^−1^ of light for 16 h and (20 °C)/8 h dark (16 °C). Transformants were identified by survival on the antibiotic selection plates (30 µg/mL kanamycin). After 7 days, seedlings were transferred to individual pots containing SunGrow professional soil mix #1 (Sun Gro Horticulture, Agawam, MA, USA) and allowed to set seeds, which were harvested when fully desiccated. In all experiments wild-type plants were untransformed Columbia-0 (Col-0). Direct PCR amplification was done with MyTaq™ Plant-PCR 2X Mix (Bioline, Memphis, TN, USA), a small (0.7 mm) fresh leaf tissue disc, and gene-specific primers to confirm the presence of the T-DNA. Sequences of all primers used are listed in Table S1. PCR products were run on a 1.5% gel at 150 V stained with SYBRSafe™ (ThermoFisher, Waltham, MD, USA). T_2_ seeds were grown on selection plates and screened for single locus insertion by calculating chi-square values to verify a ratio of 3:1 resistant: sensitive plants. The single locus insertion lines were then grown for seed and plated again. By the T_4_ generation all plants in all three lines used for further experiments were kanamycin resistant and assumed to be homozygous. All T_4_ plants were grown in growth chambers and analyzed by comparison with the Col-0 wild type plants. 

### 4.3. RNA Extraction, Reverse Transcription, and RT-qPCR Analyses

Fresh whole plant tissue from 6-week-old *A. thaliana* plants was flash frozen in liquid nitrogen and total RNA isolated using the RNeasy^®^ Plant MiniKit (Qiagen, Germantown, MD, USA). RNA samples were treated with DNase I (ZymoResearch, Irvine, CA, USA) to remove genomic contamination, followed by a clean-up step performed using the RNeasy^®^ Plant MiniKit according to manufacturer’s recommendations. RNA quality and quantity were determined using a NanoDrop ND-2000c spectrophotometer (ThermoFisher, USA). cDNA was synthesized with 100 ng of RNA using a ProtoScript^®^ First Strand cDNA Synthesis Kit (New England Biolabs, Ipswich, MA, USA). Real time quantitative PCR (q-PCR) was performed using the C1000 Touch™ ThermoCycler with the SsoAdvanced™ Universal SYBR^®^ Green Supermix following the manufacturer’s instructions (BioRad, Hercules, CA, USA). Reactions consisted of 10 µL of Sso Advanced™ Universal SYBR Green Supermix, 4 µL of 10 µM forward and reverse primers, 100 ng of cDNA template, and water to 20 µL. PCR cycling parameters included an initial denaturation for 30 s at 95 °C followed by 35 cycles of 95 °C denaturation, 60 °C annealing, and 72 °C extension. Final extension was performed at 65 °C for 5 min. All reactions were analyzed with a melt curve to determine if any genomic DNA contamination was present. All samples were normalized to the expression of *A. thaliana* Actin 8 (At1g4920) as an internal control (Nelson and Steber, 2017). Three independent transformants were used as biological replicates for each sample. Expression levels of *TsBAHD*, *DWF4, CPD*, and *BAS1* were assessed using primer pairs specific to each gene (listed in supplemental Table S1). Gene expression relative to the wild type was calculated using the 2^−ΔΔCT^ method [43] using actin 8 as a control housekeeping gene. *p*-values comparing *35S::TsBAHD* lines to wild type were calculated using Student’s t-test. Values were assigned as follows: * *p* < 0.05, ** *p* < 0.01, *** *p* < 0.001. Standard error was calculated for all samples in each group.

### 4.4. BL Dose Response Growth and Hypocotyl Measurements 

*A. thaliana* seeds were surface sterilized (as above) and plated with half strength Linsmaier and Skoog modified basal medium, 0.6% Gellan, and 1.5% sucrose [44]. Plates were supplemented with BL at concentrations of 0 nM, 10 nM, or 100 nM as described in Turk et al. [33]. After incubation in complete darkness for 4 days at 4 °C, germination was then induced by exposure to red-light at 70 mmol m^−2^ s^−1^ for 2 h at 25 °C. All plates were then transferred to the same growth chamber in either light or dark conditions for 4 days at 25 °C. White light was continuously provided (to maximize the de-etiolation response) to light grown seedlings at 80 µE. Dark treated plates were exposed to the identical conditions, but light exposure was prevented by wrapping in two layers of aluminum foil. Growth and hypocotyl measurements were performed as described by Sandhu et al. [45]. Briefly, individual seedlings were removed from plates and placed on acetate sheets where hypocotyl lengths were scanned using a flat-bed scanner at a resolution of 1200 dpi. Hypocotyl lengths were determined from the scanned images using ImageJ [46]. Hypocotyls from each group were measured, averaged, and compared using the Student’s t-test. Three independent homozygous lines were grown in two separate replicates at different times in the same growth chamber and conditions. *p*-values were calculated using Student’s t-test and standard error calculated for all samples in each group.

## Supplementary Materials

The following are available online at https://www.mdpi.com/2223-7747/9/11/1566/s1, Table S1: PCR primers.
